# Cardiovascular prognosis in patients admitted to an emergency department with hypertensive emergencies and urgencies

**DOI:** 10.1097/HJH.0000000000002961

**Published:** 2021-08-20

**Authors:** Anna Paini, Luca Tarozzi, Fabio Bertacchini, Carlo Aggiusti, Claudia Agabiti Rosei, Carolina De Ciuceis, Paolo Malerba, Alberto Broggi, Cristiano Perani, Massimo Salvetti, Maria Lorenza Muiesan

**Affiliations:** a Università di Brescia; b DEA Spedali Civili di Brescia, Brescia, Italy

**Keywords:** hypertensive emergencies, hypertensive urgencies, outcome, prognosis

## Abstract

**Background:**

At present, few data are available on the prognosis of hypertensive emergencies and urgencies admitted to emergency departments.

**Aim:**

The aim of our study was to evaluate the incidence of total and cardiovascular events during follow-up in hypertensive patients admitted to the emergency departments of Brescia Hospital (Northern Italy) with hypertensive emergencies or urgencies from 1 January to 31 December 2015.

**Methods:**

Medical records of patients aged more than 18 years, admitted to the emergency department with SBP values at least 180 mmHg (SBP) and/or DBP values at least 120 mmHg (DBP) were collected and analysed (18% of patients were classified as ‘hypertensive emergency’ and 82% as ‘hypertensive urgency’). Data in 895 patients (385 men and 510 women, mean age 70. 5 ± 15 years) were analysed; the mean duration of follow-up after admission to the emergency department was 12 ± 5 months.

**Results:**

During the follow-up, 96 cardiovascular events (28 fatal) occurred (20 cardiac events, 30 cerebrovascular events, 26 hospital admission for heart failure, 20 cases of new onset kidney disease). In 40 patients (4.5%), a new episode of acute blood pressure rise with referral to the emergency department was recorded. Cardiovascular mortality and morbidity were greater in patients with a previous hypertensive emergency (14.5 vs. 4.5% in patients with hypertensive emergency and urgency, respectively, chi-square, *P* < 0.0001). Similar results were obtained when the occurrence of cerebrovascular or renal events were considered separately.

**Conclusion:**

Admission to the emergency department for hypertensive emergencies and urgencies identifies hypertensive patients at increased risk for fatal and nonfatal cardiovascular events. Our findings add some new finding suggesting that further research in this field should be improved aiming to define, prevent, treat and follow hypertensive urgencies and emergencies.

## INTRODUCTION

Clinicians have to frequently manage acute BP reduction for a critically ill patient, with specific indications suggested by more recent guidelines or consensus papers [1–3]. Conversely, a large number of patients admitted to the emergency department have longstanding hypertension and should be referred to outpatient care rather than receive acute interventions [4,5]. The short-term and long-term effects of acute BP-lowering on cardiac and cerebrovascular morbidity and mortality have been evaluated in few clinical trials or surveys [6–14]. Data on the prevalence of patients referred to the emergency departments describing their clinical features have increased in more recent years, underlying the relevance of this topic from a public health perspective [9,13–19]. At the same time, more data are needed in the everyday management and follow-up of these patients. In particular, it is not clear whether patients with very high BP values (usually >180/110 mmHg) but absence of acute hypertension-mediated target organ damage should be considered as ‘uncontrolled hypertension’ or ‘hypertensive urgency’. As recently proposed, ‘hypertensive urgencies’ should not be considered a separate entity [3] as there is no evidence that treatment in patients who lack acute hypertension-mediated organ damage is different from patients with asymptomatic uncontrolled hypertension. Some data [9] collected in an office setting have demonstrated that cardiovascular risk is not particularly high in these patients; in addition BP control and/or the incidence of major cardiovascular events was similar in those sent home or referred to the emergency department. Other studies [10,13–15] have shown that even patients with hypertensive urgencies, in the absence of hypertension-mediated organ damage, compared with hypertensive patients with lower BP values in the office or at admission to the emergency department, have a poorer event-free survival.

On the basis of the above considerations, we considered worthwhile to investigate the occurrence of clinical events and blood pressure control during follow-up, in patients with hypertensive emergencies and hypertensive urgencies referred to the emergency department of ‘ASST Spedali Civili of Brescia’ during a period of 12 months.

## METHODS

Consecutive patients admitted to the emergency department of the Spedali Civili of Brescia aged 18 years or older and presenting with an acute increase in BP values were included in the study. The procedure was approved by the Ethics committees. The study was conducted in accordance with the Helsinki Declaration. In addition, written or oral informed consent was obtained by patient or authorized relatives. When patients could not provide the necessary information, their relatives provided the data registered. All patients (Italian citizens and foreigners) had free access to the emergency department of the Spedali Civili of Brescia, as established by the Italian National Health Service. The procedures followed were in accordance with institutional guidelines. An acute increase in BP was defined as SBP at least 180 mmHg and/or DBP at least 120 mmHg [3,17]. Women affected by eclampsia and preeclampsia or HELLP syndrome were not included in the study as they are directly referred to the Obstetrics Clinics. In addition, patients with resuscitated cardiac arrest, going directly to the cath laboratory for coronary angiography, were excluded. Each patient underwent a throughout medical examination, including clinical history, physical examination and routine blood and urine chemical analyses, if necessary, according to standardized measurements and procedures. In patients with a previous diagnosis of hypertension, medical history and current treatments were collected. BP was measured in the presence of the doctor, in the emergency room with the patient in the recumbent position by use of a mercury sphygmomanometer, according to a standard technique. When pain and anxiety were clearly evident, BP was measured after alleviation of the stressful condition. Patients with acute BP elevation were further divided as having either hypertensive emergencies or urgencies on the basis of presence or absence, respectively, of acute or progressive end-organ damage. Impending or progressive organ damage was considered in case of hypertensive encephalopathy, ischemic stroke, intracranial haemorrhage, acute coronary syndrome (acute myocardial infarction or unstable angina), acute left ventricular failure, acute pulmonary oedema, aortic dissection and progressive acute renal failure. The presence of impending or progressive organ damage was diagnosed on the basis of clinical data and diagnostic tests whenever appropriate, such as blood and urine chemistry, ECG, chest radiograph, computed tomography, ultrasound imaging and eye fundus examination. Acute aortic dissection was considered in any patient complaining of chest pain, back pain or abdominal pain associated with high values of BP, and diagnosis confirmed by computed tomography angiography. Acute coronary syndrome included the ST-elevation myocardial infarction (STEMI, ST-segment elevations of more than 0.1 mV in two corresponding leads and a typical rise and fall of cardiac enzymes), the non-STEMI (NSTEMI, considered as electrocardiographic ST-segment depression or prominent T-wave inversion and/or positive biomarkers of necrosis in the absence of ST-segment elevation and in an appropriate clinical setting, such as chest discomfort or angina equivalent) and unstable angina pectoris (ischemic symptoms suggestive of an acute coronary syndrome and no elevation in troponin or creatin kinase-MB, with or without ECG changes indicative of ischemia) with the need of coronary angiography and/or intervention. Acute pulmonary oedema was defined as evidence of clinical signs and confirmed by chest radiograph. In addition, systolic left ventricular function was assessed by a standard echocardiographic examination. Hypertensive encephalopathy was defined as progressive appearance of severe headache, nausea, vomiting and visual disorders, with or without localized or generalized seizures. Acute stroke was defined by neurological symptoms (aphasia, hemianopsia, paresthesia or paresis) lasting more than 24 h and by computer tomography of the brain revealing either ischemic or haemorrhagic area. All information on vital parameters, clinical presentation, standard laboratory examinations and treatment administration were collected from the medical records of the emergency department.

### Follow-up

Follow-up data were obtained by telephone contact in all patients. For each patient, the occurrence of cardiovascular events, including cardiac events (acute coronary syndrome, acute heart failure), cerebrovascular events (ischemic or hemorrhagic stroke or transient ischemic attack) and renal events (acute renal failure or hemodialysis) were recorded; patients were also asked to report further emergency department admission for acute BP increase. If a new hospitalization occurred, medical records were collected. In addition, the use of antihypertensive treatment and the measurement of blood pressure values less than 135/85 mmHg (i.e. BP control) were recorded.

### Statistical analysis

All data were collected and analysed by SPSS 20 (SPSS Inc, Chicago, Illinois, USA) software. Data are expressed as mean standard deviation for continuous variables and percentages for categorical variables. The differences between continuous variables were analysed by the Student's *t* test for unpaired data. Differences between categorical variables were analysed by the *χ*^2^. Kaplan--Meier survival analysis was used to assess the event-free survival in patients with hypertensive emergencies and hypertensive urgencies. The diagnosis of hypertensive emergencies or hypertensive urgencies was tested as independent variable in multivariate Cox proportional hazard model analyses adjusted for the confounders, having all or fatal cardiovascular events as dichotomic-dependent variables.

## RESULTS

In 2015, out of 69 101 patients admitted to emergency department, 1214 (1.76%) (mean age 70 + 14 years, 41% men) had a hypertensive emergency (*n* = 187, 15.4%, 0.22% of all emergency department visits) or urgency (*n* = 1027, 84.5%, 1.1% of all emergency department visits).

Out of 1214 patients assessed in the emergency department during the 12 months period in 2015, 11 patients died shortly after hospitalization (nine patients with hypertensive emergency, seven with a cerebrovascular event, one with a coronary syndrome and one with an acute heart failure) and two patients with hypertensive urgency with an advanced neoplastic disease died from nosocomial infection.

**FIGURE 1:**
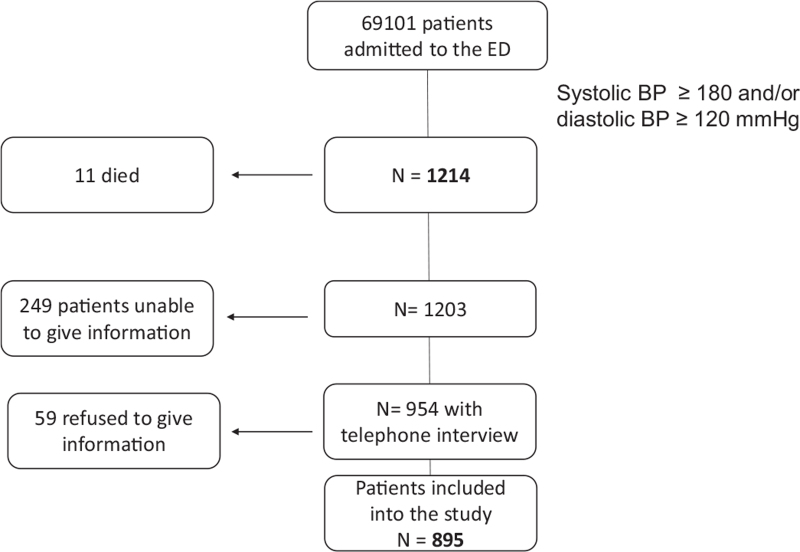
Flow-chart showing the selection of the study population.

Out of the remaining 1203 patients, 249 were not able to give information and 59 refused to answer to the interview; at the end 895 patients (43% men, mean age 70.5 + 15 years) were available for the telephone interview (Fig. 1). No significant differences in patients’ demographic characteristics, comorbidities and prevalence of hypertensive emergencies and hypertensive urgencies were observed between the whole group of patients and those included in the follow-up (Supplemental Table 1).

Prevalence of arterial hypertension and diabetes mellitus were 73 and 22%, respectively, 18% had a previous diagnosis of ischemic heart disease and 15% of cerebrovascular diseases; 737 patients (82%) presented as hypertensive urgency and 158 (18%) as hypertensive emergency.

Patients with hypertensive emergencies compared with hypertensive urgencies were older, more frequently men (56 vs. 40%), had higher prevalence of previous cardiovascular disease, and had slightly higher values of SBP and DBP at admission in the emergency department (Table 1).

**TABLE 1:** Characteristics of patients with hypertensive emergencies and hypertensive urgencies

	Hypertensive emergencies	Hypertensive urgencies	*P*
Age (years)	73 ± 13	70 ± 15	<0.05
Sex (M) (%)	55.7	39.9	<0.001
Smoke (yes) (%)	18.3	13.5	NS
History of hypertension (%)	82.9	70.5	0.01
History of diabetes (%)	27.2	20.9	<0.02
History of CAD (%)	29.7	16.7	<0.001
History of cerobrovascular disease (%)	22.8	13.3	0.02
SBP (mmHg)	192 ± 17	189 ± 12	<0.01
DBP (mmHg)	99 ± 18	93 ± 15	<0.001
HR (beats/min)	82 ± 20	80 ± 17	NS

CAD, coronary artery disease; HR, heart rate.

Predominant organ damage in the hypertensive group were heart failure (30%), acute coronary syndrome (27%), neurological disorders/stroke (37%) and acute kidney failure (6%).

During the follow-up (mean duration 12 ± 5 months), 77% of patients with hypertensive urgency and 89% of patients with hypertensive emergency was treated with different antihypertensive drugs. Among patients with hypertensive urgencies and hypertensive emergencies, no treatment during the follow-up was reported in 14.5 and 2.5% of cases (*P* < 0.001), whereas withdrawal of prescribed treatment was recorded in 3.7 and 1.9% (*P* < 0.05), respectively (Table 2).

**TABLE 2:** Treatment with cardiovascular drugs during follow-up in patients with hypertensive emergencies and hypertensive urgencies

	Hypertensive emergencies	Hypertensive urgencies	*X* ^*2*^ * P*
β blockers (%)	54	29	<0.001
ACE-inhibitors (%)	41	28	<0.001
Angiotensin receptor antagonists, ARB (%)	26	29	NS
Dihydropiridinic calcium channel blockers (%)	40	29	<0.02
Nondihydropiridinic calcium channel blockers (%)	3	2	NS
Clonidine (%)	6	2	<0.02
Doxazosin (%)	10	6	NS
Nitrates (%)	9	5	NS
Thiazide diuretics (%)	12	17	NS
Loop diuretics (%)	32	12	<0.001
Aldosterone-antagonists diuretics (%)	18	2	0.001
Mean number of drugs/patient (*n*)	2.53	1.62	<0.001

ACE, angiotensin-converting enzyme.

Twenty patients with hypertensive urgencies reported normal BP values without any treatment after the discharge from the emergency department, and therefore, withdrew the prescribed treatment, remaining normotensive, other seven patients refused treatment. Among patients with hypertensive emergencies, three with a previous stroke reported normal BP values and were not anymore treated with antihypertensive drugs.

The use of beta-blockers, of ACE inhibitors, of dihydropiridinic calcium channel blockers, of clonidine and of diuretics was greater in the group with hypertensive emergencies, possibly reflecting the higher prevalence of comorbidities (Table 3).

**TABLE 3:** Characteristics of patients with and without cardiovascular events during the follow-up

	CV events	No CV events	*P*
Sex (M, %)	52%	41%	<0.02
Age (years)	74 ± 12	69 ± 15	<0.02
Smoke (yes) (%)	24.3	14.1	NS
History of hypertension (%)	83	72	0.02
History of diabetes (%)	37.2	20.7	<0.001
History of cerobrovascular disease (%)	33	12.8	<0.001
History of CAD (%)	33	17.3	<0.001
SBP (mmHg) at first ED access	191 ± 16	189 ± 13	NS
DBP (mmHg) at first ED access	97 ± 17	94 ± 15	NS
HR (beats/min) at first ED access	82 ± 19	80 ± 17	NS
BP control during follow-up (yes) (%)	56%	78%	<0.001

CAD, coronary artery disease; CV, cardiovascular; ED, emergency department.

Concerning the follow-up, BP measurements were available in 94% of patients and a similar prevalence of BP control (76%) was reported in patients with hypertensive emergencies and hypertensive urgencies; in few patients in both groups (6%) no data on BP measurement were recorded.

In 3 cases, a diagnosis of new-onset diabetes, and in 15, a new diagnosis of hypertension was reported. In 40 patients (4.5%), a new episode of acute BP rise with referral to the emergency department was recorded, without a significant difference between hypertensive emergencies and hypertensive urgencies (3.9 vs. 3.3%, respectively, *P* = NS).

During the follow-up, 203 new events (69 deaths), requiring hospitalization, occurred: 96 cardiovascular (28 fatal) and 107 noncardiovascular (41 fatal). Among 96 cardiovascular events, 20 were cardiac ischemic events (4 deaths), 30 cerebrovascular events (16 deaths), 26 hospital admission for heart failure (6 deaths) and 20 cases of renal failure or need for haemodialysis treatment (2 deaths).

Patients with cardiovascular events during the follow-up were older, more frequently men, and with a higher prevalence of hypertension, diabetes, cerebrovascular and cardiac diseases; in addition, BP control during follow-up was higher in patients without CV events as compared to patients with CV events (78 vs. 56%, *P* < 0.01).

Morbidity and mortality for cardiac events (i.e. acute coronary syndromes and/or acute heart failure) were higher in patients with a previous hypertensive emergencies [12.9 and 3.7% in patients with hypertensive emergencies and hypertensive urgencies, respectively, *P* < 0.0001; odds ratio (OR) 3.83, 95% CI 7.03–20.9]. Similar results were obtained when the occurrence of heart failure (7.7 and 1.9% in patients with hypertensive emergencies and hypertensive urgencies, respectively; OR 3.23, 95% CI 1.3–8.05), coronary events (5.2 and 1.7% in patients with hypertensive emergencies and hypertensive urgencies, respectively; OR 3.23, 95% CI 1.3–8.05, *P* < 0.01), cerebrovascular events (11 and 1.9% in patients with hypertensive emergencies and hypertensive urgencies, respectively; OR 4.26, 95% CI 1.93–9.41) or renal events (4.5 and 1.8% in patients with hypertensive emergencies and hypertensive urgencies, respectively; OR 2.59, 95% CI 1–6.6) were considered separately. No differences were observed between patients with hypertensive emergencies and hypertensive urgencies in the occurrence of hospitalization for all noncardiovascular causes (12.9 and 11%, *P* = NS; OR 1.3, 95% CI 0.8–2.2) or non-cardiovascular mortality (5.2 and 4.6%, *P* = NS; OR 0.88, 95% CI 0.40–1.9).

**FIGURE 2:**
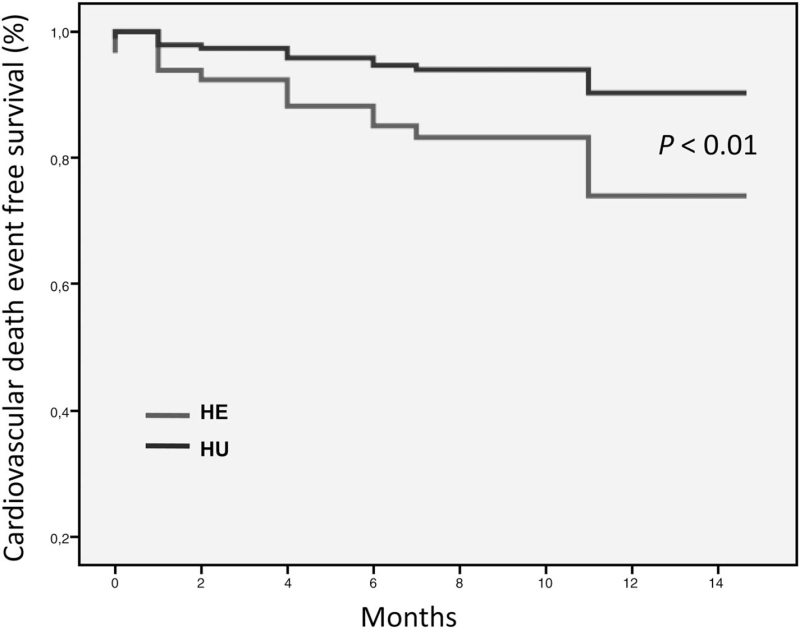
Cox regression analysis for cardiovascular death event-free survival in patients with hypertensive emergencies and hypertensive urgencies.

In the Cox regression analysis including hypertensive emergencies and hypertensive urgencies, age, sex, clinical BP values, diabetes, previous diagnosis of coronary artery disease, and previous diagnosis of cerebrovascular disease only age (hazard ratio 1.066, 95% CI 1.026–1.108, *P* = 0.001), DBP (hazard ratio 1.034, 95% CI 1.010–1.058, *P* = 0.004), previous cerebrovascular disease (hazard ratio 4.174, 95% CI 1.940–8.983, *P* < 0.001) and being an hypertensive emergency (hazard ratio 6.983, 95% CI 3.082–15.822, *P* < 0.001) were revealed to be independent predictors of subsequent cardiovascular fatal events (Fig. 2). In multivariate Cox analyses adjusted for sex, age, clinical BP, diabetes, previous diagnosis of coronary artery disease, previous diagnosis of cerebrovascular disease, hypertensive emergencies (hazard ratio 3.319, CI 2.162–15.095, *P* = 0.001), age (hazard ratio 1.018, CI 1.000–1.036, *P* = 0.047), diabetes (hazard ratio 1.686, CI 1.092–2.605, *P* = 0.019), and a previous diagnosis of a cerebrovascular disease (hazard ratio 2.234, CI 1.426–3.500, *P* = 0.001) were independent predictors of cardiovascular fatal and nonfatal events, whereas sex and clinical BP did not reach statistical significance.

## DISCUSSION

Our study provides a description of 1-year follow-up of consecutive patients referred in the emergency department of a tertiary hospital in northern Italy (Brescia) with hypertensive urgencies or hypertensive emergencies. We have shown a high 1-year cardiovascular event rate and high 1-year death rate for cardiovascular causes in both hypertensive emergencies and hypertensive urgencies.

These data expand our previous observation [17] and other studies [8–15], providing an insight into this high-risk setting of acute hypertension increase.

We have previously reported the demographic and clinical characteristics of patients examined in two different period of time (2008 and 2015) showing a prevalence of clinical presentation, which is similar to other observational studies [20,21].

We observed slight differences between hypertensive emergencies and hypertensive urgencies, with higher mean age and men prevalence for hypertensive emergencies. The difference in sex prevalence could be because of the absence of pregnant women with emergencies, whereas the older age of hypertensive emergencies could be the expression of more comorbidities like hypertension, cardiovascular disease and diabetes mellitus leading to higher degree of atherosclerosis [22,23] and further increasing the cardiovascular risk profile of these patients.

In our study, patients with hypertensive emergencies present a significantly higher cardiovascular event rate, possibly explained by their higher prevalence of previous cardiovascular disease, and to a lesser extent by differences in age or sex (being men at higher cardiovascular risk profile) or other risk factors, including the degree of BP increase during the acute episode. During follow-up, stroke was the leading cardiovascular event in our study population, followed by heart failure, renal failure and acute coronary syndromes, although the higher risk of subsequent events was confirmed in our patients with all different clinical presentation of hypertension-mediated organ damage.

BP control during the follow-up in our study was not different between hypertensive emergencies and hypertensive urgencies, reaching more than 70% among those receiving treatment. This finding is similar to the observation of Vleck *et al.*[13], showing a prevalence of BP control of 56 and 50% and comparable SBP and DBP values measured during follow-up in patients admitted with a hypertensive urgency or in the control group of hypertensive patients (admitted to the emergency department for several reasons and with BP values >140 and/or 90 mmHg). However, we cannot exclude the role of uncontrolled BP in favoring the occurrence of cardiovascular events [14] as the achievement of BP control was reported in a lower percentage of patients who developed subsequent cardiovascular events during the follow-up. The lack of differences in noncardiovascular hospitalization and death (because of neoplastic diseases, trauma or severe infections) between patients with hypertensive emergencies and hypertensive urgencies not only strongly reinforces the role of optimal control of BP but also of all other risk factors in cardiovascular secondary prevention.

A recent consensus document suggests the use of uncontrolled BP as a term in substitution of hypertensive urgency, since the management with oral antihypertensive drug administration and the outcome of these patients does not differ from those with poorly controlled BP. In fact, Patel *et al.*[9] have observed that major cardiovascular events occurred after 6 months in 0.9% of patients with BP values greater than 180/110 mmHg in the office, despite they were sent home from the hospital. On the opposite, as demonstrated by other several studies [10,13–15] the prognosis of patients admitted to an emergency department for an acute rise in BP, despite the absence of hypertension-mediated organ damage, may be associated with a high risk of a cardiovascular event. Vleck *et al.*[13] have followed 384 hypertensive urgencies with a mean age of 56 years for a mean period of 5 years and have shown a cardiovascular fatal and nonfatal events rate of 23%, corresponding, roughly to an incidence of 4.6/100 patients per year. Guiga *et al.*[10] report a 12-month mortality of 38.9% for hypertensive emergencies and 8.9% for hypertensive urgencies. Mancusi *et al.*[14] observed that the excess cardiovascular event risk in patients with hypertensive urgencies could be mediated through higher prevalence of left ventricular hypertrophy and carotid plaques. In our study, the incidence of all cardiovascular events was 27.2 and 7.2/100 patients/year, respectively in hypertensive emergencies and hypertensive urgencies, once again confirming the high cardiovascular risk of these patients, higher than that observed in clinical trials including patients at high cardiovascular risk, such as the LIFE [24] or the VALUE [25] studies (composite endpoint rate 3.2 and 2.5/100 patients/year, respectively).

Most patients with a clear clinical phenotype of hypertension-mediated organ damage are admitted to the emergency department for usually mild and aspecific symptoms like dizziness, headache, pain, frequently associated to anxiety [26]. A less aggressive approach in performing further tests for subclinical organ damage or in prescribing treatment or short-term re-evaluation by a specialist is the most common medical approach to this group of patients [19], perhaps underestimating the risk for subsequent cardiovascular events in this group of patients.

In addition, anxiety, psychopathological traits and drug nonadherence have been frequently overstated causes of hypertensive urgencies [26–28]. Very recently nonadherence to prescribed antihypertensive medication has been reported in only 25% of patients with hypertensive urgencies presenting at the emergency department [29], and was associated with male sex and with a higher number of antihypertensive drugs, but not with depression or anxiety.

In our study, treated hypertensive patients with hypertensive urgencies had a high rate of BP control during the follow-up, although a greater percentage of patients did not receive antihypertensive treatment or decided to withdraw the prescribed treatment during the follow-up. Therefore, we cannot exclude that in some patients, BP values remained elevated in the long-term, justifying, at least in part, the occurrence of events.

Our registry has some obvious limitations. First, this is an observational study, with a retrospective analysis of data prospectively collected in 2015 [17], and therefore, representing the management for hypertensive emergencies and urgencies after emergency department referral and hospitalization in a tertiary hospital in Italy 6 years ago. Second, we have not performed a follow-up visit but a telephone interview, collecting all available medical records examination from the general practitioners and by the Hospital Information System. We were able to contact 895 out of the original 1214 patients, although clinical characteristics of the original sample and of patients included in this report were superimposable. We believe that a possible consequence is the underestimation rather than overrating of cardiovascular risk in this population.

When evaluating treatment, we categorize for classes of drugs and did not record the exact doses of all drugs for management of all patients during the follow-up.

Thirdly the results cannot be generalized to other settings as these data were collected at a single department. However, several observational available studies have shown a high rate of cardiovascular events not only in patients with hypertensive emergencies but also with hypertensive urgencies.

In conclusion, we have underscored the importance of organizing comprehensive research efforts to improve current knowledge in this growing group of high-risk patients [30], hoping that in the future, we will be able to provide better overall care and improve cardiovascular outcome.

## ACKNOWLEDGEMENTS

### Conflicts of interest

There are no conflicts of interest.

## Supplementary Material

Supplemental Digital Content
