# Supplementary material for: Cardiovascular prognosis in patients admitted to an emergency department with hypertensive emergencies and urgencies
Source: J Hypertens. 2021 Aug 20;39(12):2514–20. doi: 10.1097/HJH.0000000000002961 (PMC9698186; doi:10.1097/HJH.0000000000002961)
Supplement: Supplemental Digital Content [file jhype-39-2514-s001.docx]

Supplemental table 1 Demographic and clinical characteristics of all patients and of patients included in the follow up

|  | **All**  **n = 1214** | **Follow Up**  **n = 895** | **P** |
| --- | --- | --- | --- |
| Gender (M/F) (%) | 41/59 | 44/56 | NS |
| Age (years) | 69,7±15 | 71 ± 13 | NS |
| SBP (mmHg) | 189,3±13 | 189,5±13 | NS |
| DBP (mmHg) | 94,7±15 | 94,6±15 | NS |
| HR (b/min) | 80,9±17 | 80,8±17 | NS |
| Smoke (yes) (%) | 16,5 | 15,0 | NS |
| History of hypertension (%) | 72,7 | 67 | NS |
| History of CAD (%) | 19,0 | 22 | NS |
| History of diabetes (%) | 22,4 | 24 | NS |
| History of cerobrovascular disease (%) | 14,4 | 18 | NS |
| Hypertensive emergency (%) | 15,5 | 18 | NS |
| Hypertensive urgency % | 84.5 | 82 | NS |
